# Decoding the Physical Mechanisms of Protein-Small Molecule Association by Multiscale Kinetic Monte-Carlo Simulations

**DOI:** 10.64898/2026.10.23.761358

**Published:** 2026-10-25

**Authors:** Zhaoqian Su, Yinghao Wu

## Abstract

Protein-small molecule association involves multiple recognition stages governed by distinct physical interactions, yet resolving their kinetic contributions remains computationally challenging. Here, we develop a multiscale Monte Carlo framework that combines an atomistic, conformationally flexible ligand with a coarse-grained protein binding pocket and an atomistic steric envelope. Association is decomposed into three stages: molecular encounter, pocket entry, and productive binding. We evaluate the framework using ten protein–ligand complexes representing nine distinct targets, with experimentally derived association rates spanning nearly four orders of magnitude. Simulated pocket-entry rates exhibit the strongest correspondence with experimental kinetics (Pearson r = 0.617; Spearman ρ = 0.758), whereas productive-binding rates show closer agreement in absolute magnitude. Systematic perturbations of electrostatic interactions, hydrophobic attraction, hydrogen bonding, and ligand torsional flexibility reveal stage-dependent sensitivities. Initial encounter is comparatively insensitive to these perturbations, while hydrophobic attraction and conformational flexibility influence pocket entry. Productive binding exhibits additional sensitivity to local interactions, with excessive hydrogen-bond strengthening potentially hindering native-pose formation. Probability-derived kinetic-stage barriers and normalized mechanism descriptors further reveal heterogeneous recognition behavior across protein targets. Together, this framework provides a computationally efficient and interpretable approach for investigating protein-ligand association pathways and their physical determinants across diverse molecular systems.

